# Transcriptomic and lipidomic profiling of subcutaneous and visceral adipose tissues in 15 vertebrates

**DOI:** 10.1038/s41597-023-02360-3

**Published:** 2023-07-12

**Authors:** Pengliang Liu, Diyan Li, Jiaman Zhang, Mengnan He, Yan Li, Rui Liu, Mingzhou Li

**Affiliations:** 1 School of Pharmacy, Chengdu University, Chengdu, 610106 China; 2 Livestock and Poultry Multi-omics Key Laboratory of Ministry of Agriculture and Rural Affairs, College of Animal Science and Technology, Sichuan Agricultural University, Chengdu, 611130 China; 3 Animal Breeding and Genetics Key Laboratory of Sichuan Province, Institute of Animal Genetics and Breeding, Sichuan Agricultural University, Chengdu, 611130 China; 4 Chengdu Research Base of Giant Panda Breeding, Chengdu, 611081 China

**Keywords:** Evolutionary genetics, Transcriptomics, Evolutionary biology, Lipidomics

## Abstract

The storage of lipids as energy in adipose tissue (AT) has been conserved over the course of evolution. However, substantial differences in ATs physiological activities were reported among species. Hence, establishing the mechanisms shaping evolutionarily divergence in ATs transcriptomes could provide a deeper understanding of AT regulation and its roles in obesity-related diseases. While previous studies performed anatomical, physiological and morphological comparisons between ATs across different species, little is currently understood at the molecular phenotypic levels. Here, we characterized transcriptional and lipidomic profiles of available subcutaneous and visceral ATs samples across 15 vertebrate species, spanning more than 300 million years of evolution, including placental mammals, birds and reptiles. We provide detailed descriptions of the datasets produced in this study and report gene expression and lipid profiles across samples. We demonstrate these data are robust and reveal the AT transcriptome and lipidome vary greater among species than within the same species. These datasets may serve as a resource for future studies on the functional differences among ATs in vertebrate species.

## Background & Summary

The adipose tissue (AT) is one of the most important organs ensuring energy and metabolic homeostasis in vertebrates^1^. In recent years, AT has gained sustained scientific attention due to the significant increase in global rates of obesity and metabolic disorders in human populations, in particular type II diabetes and cardiovascular disease. Recent studies showed that the AT is a remarkably complex organ playing important roles in energy storage, pathophysiology, and a variety of biological processes, such as controlling blood pressure, reproduction and host defense^2,3^. The AT is distributed across the body^4^ and can be divided into the intra-abdominal visceral AT (VAT) – located around the omentum, intestines, gonad, pericardium and perirenal areas, and the subcutaneous AT (SAT) – located in the buttocks, thighs, and abdomen. ATs from different locations have distinct properties, including different metabolic functions, structural roles or association with diseases^5–8^.

A previous study suggested that the root of AT complexity emerged during the course of evolution^9^ due to differences in AT properties between species^10^, which can be evaluated by performing cross-species comparisons. A recent comparison between humans and mice identified different proportions of a subpopulation of adipocytes regulating thermogenesis between the two species^11^, partly explaining observed differences in thermogenic activity. Moreover, well-documented comparative transcriptome analyses across phylogenies can advance translational medicine by identifying novel therapeutic targets^12^. For example, a former study found that miR-26a, a microRNA involved in cardiomyocyte proliferation, is down-regulated in injured zebrafish hearts but remains constant in mice^12^. The knockdown of miR-26a in post-natal mice hearts prolonged the proliferative window of cardiomyocytes, indicating this miRNA could be a therapeutic target for treating heart damaged^12^. Accordingly, evaluating AT changes at the molecular-level across species will enhance our understanding of the function and genetic basis of AT and its association with different diseases.

**Fig. 1:**
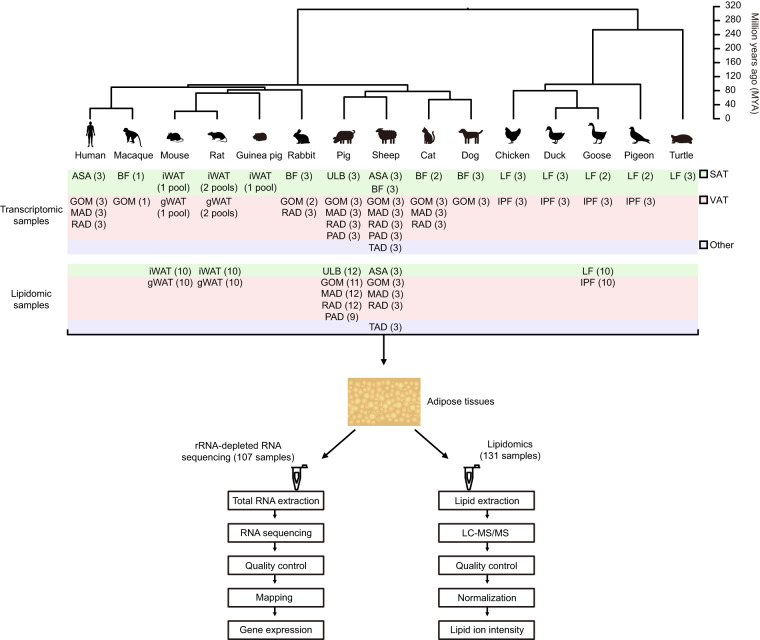
Graphical representation of the study design and data processing. Phylogenetic tree of the 15 vertebrate species used in this study, which was obtained from the Timetree database (MYA: million years ago). The number of biological replicates of each tissue are shown in parentheses. Each transcriptomic sample of mouse, rat and guinea pig represents a pool of RNAs from 10 individuals. ASA: abdominal subcutaneous adipose; BF: back fat; iWAT: inguinal white adipose tissue; LF: leg fat; ULB: upper layer of backfat; GOM: greater omentum; gWAT: gonadal white adipose tissue; IPF: intraperitoneal fat; MAD: mesenteric adipose; PAD: pericardial adipose; RAD: retroperitoneal adipose; TAD: tail adipose; SAT: subcutaneous adipose tissue; VAT: visceral adipose tissue; LC-MS/MS: liquid chromatography-tandem mass spectrometry.

Transcriptional information is important to elucidate AT phenotypes and function, but so far most studies only focused on AT comparisons between humans and rodents^13–15^. Importantly, large-scale comparative AT transcriptomic analysis across various distantly-related species and multiple anatomical locations is necessary to fully understand AT transcriptomic evolution. To this purpose, we performed a comparative transcriptomic analysis of available subcutaneous and/or visceral AT across 15 vertebrate species and locations (from 1 to 7 per species) (Fig. 1, Supplementary Table 1), including 10 mammals (primates: human [*Homo sapiens*] and macaque [*Macaca mulatta*]; rodents: mouse [*Mus musculus*], rat [*Rattus norvegicus*], and guinea pig [*Cavia porcellus*]; lagomorphs: rabbit [*Oryctolagus cuniculus*]; artiodactyls: pig [*Sus scrofa*] and sheep [*Ovis aries*]; and carnivores: cat [*Felis catus*] and dog [*Canis lupus familiaris*]), 4 birds (galliformes: chicken [*Gallus gallus*]; anseriformes: duck [*Anas platyrhynchos*] and goose [*Anser anser*]; and columbiformes: pigeon [*Columba livia*]), and one reptile (testudines: turtle [*Pelodiscus sinensis*]) as the outgroup. We generated a total of 59 paired-end rRNA-depleted RNA-seq libraries, and analyzed in combination with 48 libraries that were published previously^16–21^, totaling 107 libraries (Fig. 1, Supplementary Table 1). The lipidome composition of the ATs can impact multiple aspects of energy homeostasis, such as glucose and lipid metabolism, substrate availability and energy expenditure^22–25^. Accordingly, understanding the differences in lipid composition between ATs is essential for studying their specialized functions and exploring the potential mechanisms leading to AT heterogeneities. Lipidomics has been successfully applied previously for clarifying lipid profile changes of ATs after various treatments (such as endurance exercise training^26^, cold exposure^27^ and high-fat diet^28^) or between different anatomical locations^29^. However, changes across species remain poorly understood. To gain further insights into the metabolic changes that occurred during AT evolution, we performed untargeted liquid chromatography-tandem mass spectrometry (LC-MS/MS) analysis of the cellular lipidome of 131 SAT and VAT samples across five representative species, including four mammals (mouse, rat, pig, sheep) and a bird (goose) (Fig. 1, Supplementary Table 2). Altogether, these datasets provide a valuable resource for the study of AT genetic and metabolic diversity across species and anatomical locations, and an unprecedented opportunity to analyze molecular changes during AT evolution.

## Methods

### Ethics statement

All research involving animals in this study were conducted in accordance with the guidelines of the Administration of Affairs Concerning Experimental Animals established by the Ministry of Science and Technology of China. The experiments were approved by the Institutional Animal Care and Use Committee of the College of Animal Science and Technology, Sichuan Agricultural University, Sichuan, China under permit No. DKY-2019102006.

### Sample collection and RNA library preparation

The 107 adipose tissue samples (Fig. 1) were obtained from various sources, including 48 previously published samples^16–21^ (Supplementary Table 1), and collected from 1–20 healthy adults from each of the 15 species. All species are represented by female individuals, except for the humans (all individuals are males). Animal health was evaluated daily by a specialized laboratory animal technician (and veterinarian, if necessary). The anatomical locations of these adipose tissues are: (1) Subcutaneous adipose tissues: Abdominal subcutaneous adipose (ASA) of humans and sheep were taken from the abdominal wall, specifically from the area adjacent to the umbilicus in humans and the ventral lower abdominal area close to the mid-line in sheep; Back fat (BF) from macaque, rabbits, pigs, sheep, cats and dogs refers to the subcutaneous adipose tissue located in the mid-lower part of the back, near the dorsum midline. We note that, since the back fat of adult pigs usually has two distinct layers, we only collected the upper layer (close to skin, annotated as ULB in Fig. 1); Inguinal white adipose tissue (iWAT) of mice, rats and guinea pigs refers to the subcutaneous adipose tissue obtained from the inguinal region of both hindlimbs; Leg fat (LF) of chickens, ducks, geese, pigeons and turtles is the subcutaneous adipose tissue collected from the leg area, specifically from the upper portion of the legs (proximal to the pelvic bone) in chickens, ducks, geese and pigeons, and the dorsal base of the hind limbs in turtles. (2) Visceral adipose tissues: Greater omentum (GOM) from humans, macaque, rabbits, pigs, sheep, cats and dogs refers to an apron like structure that extends from the greater curvature of the stomach and the proximal part of the duodenum; Mesenteric adipose (MAD) of humans, pigs, sheep and cats was collected from the mesentery adjacent to the intestines; Retroperitoneal adipose (RAD) of humans, rabbits, pigs, sheep and cats is located behind the kidney, and does not contain fat surrounding the kidney; Gonadal white adipose tissue (gWAT) from the mice and rats (all individuals are females) is located around the ovary; Pericardial adipose (PAD) of pigs and sheep is located between the epicardium and parietal pericardium; Intraperitoneal fat (IPF) of chickens, ducks, geese and pigeons refers to the adipose tissue attached to the gizzard. (3) Other type adipose tissues: Tail adipose (TAD) of sheep was taken from the tail base (5–7 caudal vertebrae).

The animals generated in this study for RNA-seq were commercially obtained (the mice, rats, rabbits, sheep, cats and dogs were obtained from the Chengdu dossy experimental animals Co., Ltd, Chengdu, China; macaque and pigs were purchased from the Sichuan Hengshu Bio-Technology Co., Ltd, Yibin, China; pigeons were obtained from the Fengmao Meat Pigeon Breeding Professional Cooperative In Fucheng District of Mianyang City, Mianyang, China; chickens were purchased from the poultry breeding farm of Sichuan Agricultural University, Ya’an, China; ducks and geese were obtained from the waterfowl breeding center of Sichuan Agricultural University, Ya’an, China; and turtles were purchased from the Sichuan Longhu Lohas Turle Industry Ltd, Meishan, China), and humanely sacrificed to ameliorate suffering, in accordance with the national regulations for the care and use of research animals. All samples were immediately homogenized in liquid nitrogen and stored at −80 °C until RNA extraction was performed.

Total RNAs were extracted using the standard protocol of the RNeasy Mini Kit (Qiagen, Valencia, CA, USA). RNA quality was assessed using the Agilent 2100 Bioanalyzer (Agilent Technologies). Only samples with high quality RNA (RNA integrity [RIN] score > 7) were used for sequencing. The RNA-seq libraries were then generated using an rRNA depletion method^18^. All libraries were sequenced on a HiSeq X Ten platform (Illumina) using paired-end sequencing reads of 150 bp in length.

### RNA-seq data processing

Paired-end reads were aligned to the corresponding reference genomes (human: GRCh38.p13, macaque: Mmul_8.0.1, mouse: GRCm38.p6, rat: Rnor_6.0, guinea pig: Cavpor3.0, rabbit: OryCun2.0, pig: Sscrofa11.1, sheep: Oar_v3.1, cat: Felis_catus_9.0, dog: CanFam3.1, chicken: GRCg6a, duck: CAU_duck1.0, and turtle: PelSin_1.0; goose^30^ and pigeon [GenBank: WOFI01000000] reference genomes were housely constructed) using the STAR alignment tool (2.5.3a)^31^ with default parameters. Transcriptional abundance of protein coding genes (PCGs) was estimated as transcripts per million (TPM) using the high-speed transcript quantification tool Kallisto (0.44.0)^32^. The gene annotation file was downloaded from Ensembl (Release 89). We considered a gene as transcribed if its expression value was > 0.5 TPM in all replicates of at least one AT.

### Identification of single-copy orthologous genes

The single-copy orthologous PCG families in the 15 species were identified according to the Ensembl-recommended protocol (http://asia.ensembl.org/info/genome/compara/homology_method.html). Briefly, for each species, we extracted the longest translation of each PCG, and subsequently performed all-against-all blast between self and non-self-species. Based on the blast results, we generated a sparse graph and extracted the clusters using *hclust_sg*. The clusters with over 400 PCGs were recursively split into smaller ones until all clusters contained fewer than 400 PCGs. For each cluster, we performed multiple alignments of protein-coding sequences and back-translated to coding sequence alignments. Finally, we constructed a phylogenetic tree and identified single-copy orthologous gene families.

### Untargeted lipidomics sample preparation and LC-MS/MS analysis

We chose the SAT and VAT samples of four mammals (mouse, rat, pig, sheep) and a bird (goose) to investigate AT lipidome divergence across species (Supplementary Table 2). All of the animals are healthy adult females, and they were obtained in the same way as the RNA-seq study mentioned earlier. The specific sample information are as follows: (1) subcutaneous adipose tissue: iWAT from mice and rats, ULB from pigs, ASA from sheep, and LF from geese. (2) visceral adipose tissue: gWAT from mice and rats, GOM, MAD, RAD and PAD from pigs, GOM, MAD and RAD from sheep, and IPF from geese. (3) other type adipose tissue: TAD from sheep. The anatomical locations of these adipose tissues were detailed in ‘***Sample collection and RNA library preparation’*** above.

Total lipid extraction was performed according to a previously described method^33^ with some modifications. Briefly, 25 mg of adipose tissue was added in 800 μL precooled dichloromethane/methanol (3:1, v/v). After this, 10 μL of internal lipid standards (SPLASH Lipidomix, Avanti Polar Lipids, USA) and two steel balls were added. The mixture was homogenized using a TissueLyser for 4 min at 55 Hz. After incubation for 2 h at −20 °C, the mixture was centrifuged at 30,000 × g for 20 min at 4 °C. The 500 μL of supernatant was transferred to a new centrifuge tube and dried using a freeze-dryer. After this, 500 μL of reconstitution solvent (isopropanol/acetonitrile/water = 2:1:1, v/v/v) was added and centrifuged at 25,000 × g for 20 min at 4 °C. Next, 100 μL of supernatant was transferred to a new centrifuge tube containing 500 μL of reconstitution solvent. 80 μL of each sample was then transferred to the LC/MS vial. In addition, to monitor system stability, a quality control (QC) sample was prepared by combining the same volume of all experimental samples.

The lipid extracts were analyzed using ultra-performance liquid chromatography-tandem mass spectrometry (UPLC-MS/MS) (Waters, Manchester, UK). Each sample (5 μL) was injected onto a CSH C18 column (2.1 × 100 mm, 1.7 μm, Waters), which was kept at 55 °C with an elution rate of 0.4 mL/min. The mobile phase A was ACN: H_2_O (60:40 v/v) and the mobile phase B, was IPA: ACN (90:10 v/v), both containing 0.1% formic acid and 10 mM ammonium formate. The gradient elution conditions were: 0–2 min, 40–43% phase B; 2.1–7 min, 50–54% phase B; 7.1–13 min, 70–99% phase B; 13.1–15 min, 40% phase B. The Xevo G2-XS QTOF mass spectrometer (Waters, UK) was used to detect the metabolites eluted from the chromatographic column in both positive and negative ion modes. The positive or negative ionization mode parameters were as follows: capillary voltage 3 (pos)/2 (neg) kV; cone voltage 40 V; source temperature 120 °C; deconvolution temperature 450 (pos)/350(neg) °C. The mass spectrometry data were acquired in Continuum MS^E^ mode. The scanned *m/z* range of MS signal varied between 100 to 2000 Dalton in the positive ion mode, and 50 to 2000 Dalton in the negative ion mode. The survey scan time was 0.2 s. Based on the precursor ion intensity, the top 3 ions were selected for MS2 analysis. For the MS/MS detection, the precursors were fragmented using 19–45 eV with a scan time of 0.2 s. The leucine enkephalin signal was measured every 3 s during acquisition to calibrate the mass accuracy.

### LC-MS/MS data processing

The raw data from the mass spectrometer was imported into the commercial software Progenesis QI (version 2.2, Nonlinear dynamics, http://www.nonlinear.com/progenesis/qi) for peak detection and alignment. After this, the peak intensity data were further processed according to a previously described metaX pipeline^34^ with slight modifications. Firstly, we retained the high-quality ions present in > 50% of QC samples and > 20% of the replicates of at least one AT. The missing values were imputed using the k-nearest neighbor (KNN) method^35,36^. Next, the probabilistic quotient normalization (PQN) method^37^ was performed for data normalization and the QC-robust spline batch correction (QC-RSC) method^38^ applied to correct for batch effects. Finally, the ions with a relative standard deviation (RSD) > 30% in QC samples were removed, as are fluctuated greatly in the experiment. The retained ions were used in downstream statistical analysis.

### Statistical analysis

All statistical analyses were performed using the Wilcoxon rank-sum test in R (version 4.0.0).

## Data Records

The rRNA-depleted RNA-seq data generated in this study are available in the NCBI SRA database under accession number SRP398585^39^.

Raw lipidomics data files from each ionization mode (positive and negative) were deposited at the MetaboLights database with the accession number MTBLS5943^40^. Each MetaboLights entry contains protocols about sample collection, extraction, chromatography, mass spectrometry, metabolite identification, and data transformation.

Other data that support the findings in ‘Technical Validation’ have been deposited in the Figshare^41–44^ repository: (1) Detailed information on single-copy orthologous PCGs across 15 vertebrate species^41^; (2) PCA plot of PC1 versus PC3 and PC2 versus PC3 based on the expression levels of single-copy orthologous PCGs among 15 vertebrate species^42^; (3) Comprehensive information of all identified lipid metabolites^43^; (4) Identified lipid metabolites in the negative and positive ion modes^44^.

## Technical Validation

### Transcriptomic data

**Fig. 2:**
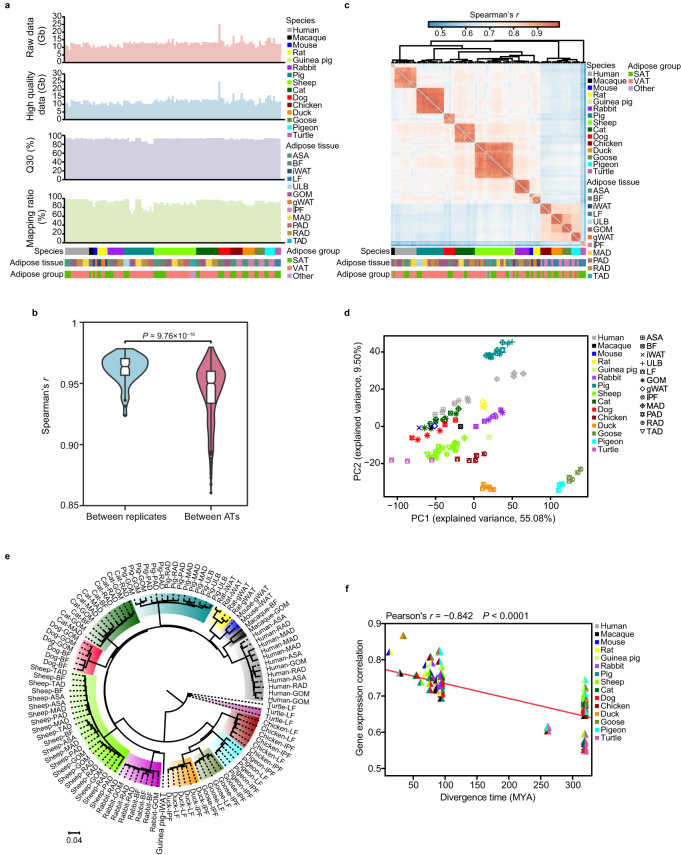
Technical validation of the transcriptomic data. (**a**) Overview of the RNA-seq data. Raw data information (first panel), high-quality data (second panel), Q30 (third panel) and mapping ratio (fourth panel) for each RNA-seq library. (**b**) Distributions of the pairwise Spearman’s correlation coefficients between biological replicates (blue) and between samples from different ATs within species (red). The line in the box indicates median, the bottom and top of colored box indicates the 25th and 75th percentiles, respectively, and the whiskers extend to 1.5 IQR from the quartiles. Each box-plot is surrounded by a violin plot displaying the distribution of data. The *P* value was determined using a Wilcoxon rank-sum test. (**c**) Hierarchical clustering analysis of 107 RNA-seq samples using the expression levels of single-copy orthologous PCGs among 15 vertebrates. Average linkage hierarchical clustering was performed following Spearman’s distances between samples using the Multiple Experiment Viewer (MEV) software^48^. (**d**) Factorial map of the principal component analysis (PCA) of the expression levels of single-copy orthologous PCGs among 15 vertebrate species. The proportion of variance explained by each principal component is provided in parentheses along each axis. (**e**) Neighbor-joining tree based on pairwise distance matrices (1−*r*, *r* is Spearman’s correlation coefficient) between expression levels of single-copy orthologous PCGs. The scale bar indicates distances. (**f**) Pairwise Spearman’s correlation coefficients of expression levels of single-copy orthologous PCGs between species were plotted against the evolutionary distance. The *P* value was calculated using hypothesis testing.

The quality of the RNA-seq data for each sample is shown in Fig. 2. Briefly, a total of ~1.36 terabases (Tb) of raw data were obtained, which is approximately 12.69 Gb per sample on average (Fig. 2a and Supplementary Table 1). The base quality of the sequencing reads was confirmed using FastQC, and consisted of a high-quality score Q30 (base error <0.1%) (median Q30 = 92.81%) (Fig. 2a and Supplementary Table 1). After filtering reads for quality and length, we retained a total of ~1.32 Tb of high-quality data, with an average of 12.30 Gb per sample (Fig. 2a and Supplementary Table 1). This allowed us to map an average of 91.64% high-quality reads to the respective genomes of the different species (Fig. 2a and Supplementary Table 1), indicating the reliability of the sequencing data.

Using a detection threshold of >0.5 TPM across all replicates in at least one AT to identify transcribed PCGs for each species, we observed comparable amounts of transcribed PCGs between mammal (~12,324 per species) and bird species (~11,973 per species) (Table 1). However, remarkably few transcribed genes were detected in the ATs of turtle (4037 PCGs), implying the AT transcriptomes of mammals and birds vary significantly from reptiles. To assess the reproducibility of the different biological replicates, we calculated pairwise Spearman’s *r* of PCGs expression profiles between the samples of each species. In general, gene expression was highly correlated between biological replicates (median Spearman’s *r* = 0.96), and similarities significantly reduced between the ATs obtained from different anatomical locations within each species (*P* = 9.76 × 10^−16^, Wilcoxon rank-sum test) (Fig. 2b). Principal component analysis and hierarchical clustering of the expression levels of 3878 single-copy orthologous PCGs (detailed information is list in file ‘Detailed information on single-copy orthologous PCGs across 15 vertebrate species’ on Figshare^41^) among 15 vertebrates revealed that samples primarily clustered according to the species with the highest number of biological replicates (Fig. 2c,d and figure **‘**PCA plot of PC1 versus PC3 and PC2 versus PC3 based on the expression levels of single-copy orthologous PCGs among 15 vertebrate species’ on Figshare^42^). These results highlight the consistency among biological replicates and the robustness of experimental design.

**Table 1:** Number of transcribed PCGs identified in each species.

Taxonomic groups	Species	Transcribed PCGs
Mammals	Human	13,127
Mammals	Macaque	12,299
Mammals	Mouse	14,644
Mammals	Rat	13,854
Mammals	Guinea pig	11,762
Mammals	Rabbit	11,815
Mammals	Pig	14,793
Mammals	Sheep	11,603
Mammals	Cat	11,086
Mammals	Dog	9036
Birds	Chicken	10,765
Birds	Duck	10,660
Birds	Goose	13,354
Birds	Pigeon	13,116
Reptiles	Turtle	4037

Finally, to validate evolutionary signatures, we conducted neighbor-joining (NJ) tree analysis based on the expression levels of 3825 transcribed single-copy orthologous PCGs (Fig. 2e), which was highly consistent with the phylogenetic tree downloaded from the Timetree database (http://www.timetree.org/) (Fig. 1). Moreover, we observed that transcriptional conservation decreases with evolutionary distance between the species (Fig. 2f), again confirming the reliability of the evolutionary signature uncovered in our dataset.

### Lipidomic data

**Fig. 3:**
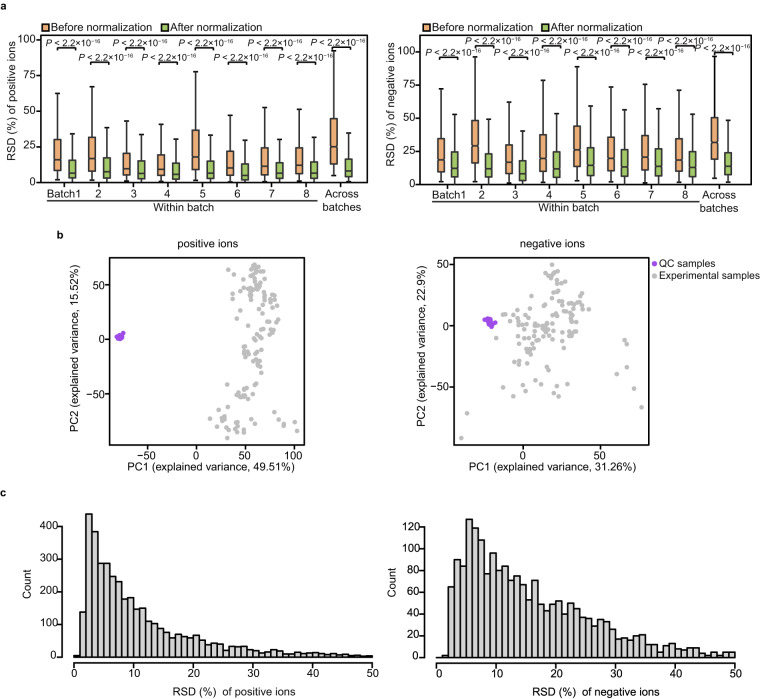
Technical validation of the lipidomics data. (**a**) Relative standard deviation (RSD) for detected ions between QC samples within the same batch or across different batches in positive (left panel) and negative (right panel) ion modes before (orange) and after normalization (green). The line in the box indicates median, the bottom and top of colored box indicates the 25th and 75th percentiles, respectively, and the whiskers extend to 1.5 IQR from the quartiles. *P* values were determined using the Wilcoxon rank-sum test. (**b**) The PCA plots of QC (purple) and experimental (grey) samples in the positive (left panel) and negative (right panel) ion modes. (**c**) The distribution of RSD for ion intensity of the compounds in the QC samples in positive (left panel) and negative (right panel) ion modes.

Lipids concentrations in 131 ATs samples obtained from five vertebrate species were analyzed using LC-MS/MS in positive and negative ion modes. After normalizing the bias within and between batches using the QC-RSC method^38^ (Fig. 3a), a total of 2652 negative ions and 4530 positive ions were detected in >50% of the QC samples (102 QC samples in negative ion mode and 96 QC samples in positive ion mode) and >20% of the experimental samples in at least one AT. To ensure the reliability of the acquired lipidomics data, two quality control steps were performed based on the intensity of the detected ions. First, we assessed the clustering of QC samples using principal component analysis (PCA). In both ion modes, we observed that the QC samples clustered distinctively (Fig. 3b), suggesting absence of significant non-biological induced variation in the experiment. Second, we measured the relative standard deviation (RSD) of detected ions in all QC samples (Fig. 3c). We found that most of the negative (67.42%, 1788 of 2652) and positive ions (81.15%, 3676 of 4530) had an RSD < 30% among the QC samples, indicating ion intensity changed little between QC samples. Overall, these findings confirmed the reproducibility and robustness of the generated lipidomics data.

**Fig. 4:**
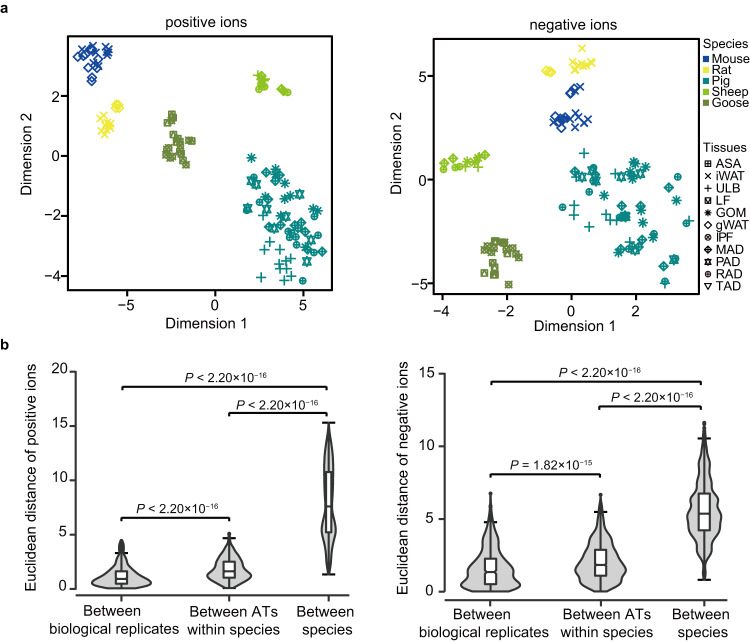
Assessment of the similarity between lipidomics samples. (**a**) *t*-distributed stochastic neighbor embedding (*t*-SNE) showing the overall variance of the ion intensity of compounds among experimental samples in positive (left panel) and negative (right panel) ion modes. Dimensions 1 and 2 were obtained from the *t*-SNE algorithm and represent distances (i.e., similarity) between samples. Notably, based on the *t*-SNE results, six rat samples were classified as “outliers” and excluded from subsequent analysis. (**b**) The Euclidean distances of ion intensity between samples in positive (left panel) and negative (right panel) ion modes, which derived from the 2D *t*-SNE plots. In each box, the central line indicates median, the bottom and top of box indicates the 25th and 75th percentiles, respectively, and the whiskers extend to 1.5 IQR from the quartiles. *P*-values were calculated using a Wilcoxon rank-sum test.

To further characterize lipid patterns and evaluate the presence of abnormal samples, we performed *t*-distributed stochastic neighbor embedding (*t*-SNE) analysis (Fig. 4a) based on the intensity of ions with RSD <30% among the QC samples, and observed a clear separation between distinct species. We next calculated the distance between samples using *t*-SNE to quantify sample variance, which also revealed that biological replicates tend to cluster close to each other. Moreover, we found that smaller distances between ATs within a species and higher between species (Fig. 4b), highlighting large variation between different species.

Finally, we annotated the ions and identified a total of 868 and 293 lipid metabolites in the positive and negative ion modes, respectively (details in file ‘Comprehensive information of all identified lipid metabolites’ on Figshare^43^). These lipid-related metabolites were part of seven lipid categories, including Fatty Acyls (FA), Glycerolipids (GL), Glycerophospholipids (GP), Prenol lipids (PR), Saccharolipids (SL), Sphingolipids (SP), Sterol Lipids (ST) (details in ‘Identified lipid metabolites in the negative and positive ion modes’ on Figshare^44^).

## Usage Notes

The AT transcriptomes across the vertebrate species used in this study were profiled using an rRNA-depleted protocol, which enables the capture of different RNA species and is more efficient in quantifying linear non-poly(A) transcripts and circular RNAs^45,46^. This will enable the investigation of dynamic PCGs expression across species, but also comparative analysis of regulatory non-protein coding transcripts (such as long non-coding RNAs) between ATs in vertebrates.

Notably, both the AT transcriptome and lipidome varied more between than within species, which highlights the functional differences that occurred over the course of evolution. This large-scale dataset encompasses a variety of species with remarkably divergent phylogenetic relationships and ATs with highly diverse anatomical distributions, documenting more than 300 million years of AT evolutionary history. This valuable resource will make it possible to understand the molecular basis of these functional differences. Theoretically, cross-species comparisons should be conducted using adipose tissues with similar anatomical locations across species. However, it is not possible to match all adipose tissues between different species. For example, there is no human equivalent of murine gonadal adipose tissue (the most commonly used visceral adipose tissue in mouse studies). This calls for omics-based comparative analysis to explore how the adipose pads of one species may correspond to the adipose depots of another species. Indeed, the assessment of human–animal relationships is important for translational studies. We believe that our data will also help to identify which species should be used in specific settings or as an animal model.

For the vast majority of the analyzed species, the AT samples were derived from multiple anatomical locations, which can be divided into SAT and VAT, allowing for evaluation of transcriptomic and lipidomic similarities and differences among ATs within species, including lesser characterized non-model organisms. They could also provide an opportunity to investigate the differences between AT locations from an evolutionary point of view. Finally, our multi-omics data can facilitate more precise investigations of the interaction between mRNAs, non-coding transcripts and metabolites across vertebrate ATs or within species across AT locations.

## Supplementary information

Supplementary table 1. Summary of all RNA-seq data across 15 vertebrate species.

Supplementary table 2. Summary of the samples used in the lipidomics study.

## Data Availability

The following software and versions were used for quality control and data processing: (1) RNA-seq data processing: read mapping was performed with the STAR alignment tool (2.5.3a)^31^; quantification of RNA-seq data was performed using Kallisto (0.44.0)^32^; identification of single-copy orthologous genes was performed using OrthoMCL^47^. (2) Lipidomics data processing: the raw data were processed using the Progenesis QI software (version 2.2, Nonlinear dynamics) for peak detection and alignment; The metaX software^34^ was further used to process peak intensity data. Associated codes have been submitted in GitHub (https://github.com/JiamanZhang/Lab_cross_15_vertebrates_adiposes_papre_code).
